# Supplementary material for: Formation of Metal-Oxide Nanocomposites with Highly Dispersed Co Particles from a Co-Zr Powder Blend by Mechanical Alloying and Hydrogen Treatment
Source: Materials (Basel). 2023 Jan 26;16(3):1074. doi: 10.3390/ma16031074 (PMC9920757; doi:10.3390/ma16031074)
Supplement: Supplementary file 1 [file materials-16-01074-s001.zip › materials-2145632-supplementary.pdf]

materials

MDPI

Supplementary Materials

Formation of Metal-Oxide Nanocomposites with Highly Dispersed Co Particles from a Co-Zr Powder Blend by Mechanical Alloying and Hydrogen Treatment

Ilya Yakovlev [1], Serguei Tikhov [1,*], Evgeny Gerasimov [1], Tatiana Kardash [1], Konstantin Valeev [1], Aleksei Salanov [1], Yurii Chesalov [1], Olga Lapina [1], Oleg Lomovskii [2] and Dina Dudina [2,3]

1 Boreskov Institute of Catalysis SB RAS, Lavrentyeva Ave. 5, 630090 Novosibirsk, Russia
2 Lavrentyev Institute of Hydrodynamics SB RAS, Lavrentyeva Ave. 15, 630090 Novosibirsk, Russia
3 Institute of Solid State Chemistry and Mechanochemistry SB RAS, Kutateladze St. 18, 630128 Novosibirsk, Russia
* Correspondence: tikhov@catalysis.ru (S. T.); Tel.: +7-3833308763

Citation: Yakovlev, I.; Tikhov, S.; Gerasimov, E.; Kardash, T.; Valeev, K.; Salanov, A.; Chesalov, Y.; Lapina, O.; Lomovskii, O.; Dudina, D. Formation of Metal-Oxide Nanocomposites with Highly Dispersed Co Particles from a Co-Zr Powder Blend by Mechanical Alloying and Hydrogen Treatment. *Materials* **2023**, *16*, 1074. https://doi.org/10.3390/ma16031074

Academic Editor: Joan-Josep Suñol

Received: 22 December 2022
Revised: 13 January 2023
Accepted: 19 January 2023
Published: 26 January 2023

Copyright: © 2023 by the authors. Licensee MDPI, Basel, Switzerland. This article is an open access article distributed under the terms and conditions of the Creative Commons Attribution (CC BY) license (https://creativecommons.org/licenses/by/4.0/).

The two-dimensional ^{59}Co Internal Field NMR experiment (sweeps both in frequency and RF power) allow discerning between single-domain and multidomain Co structures by the difference in signal enhancement factros of these structures. In Figure S1 (top), such optimal RF magnetic field is shown in arbitrary units. Figure S1 (bottom) demonstrates the results of decomposition of the experimental ^{59}Co IF NMR spectrum of the CoZr sample after hydrogen treatment into lines corresponding to different Co structures.

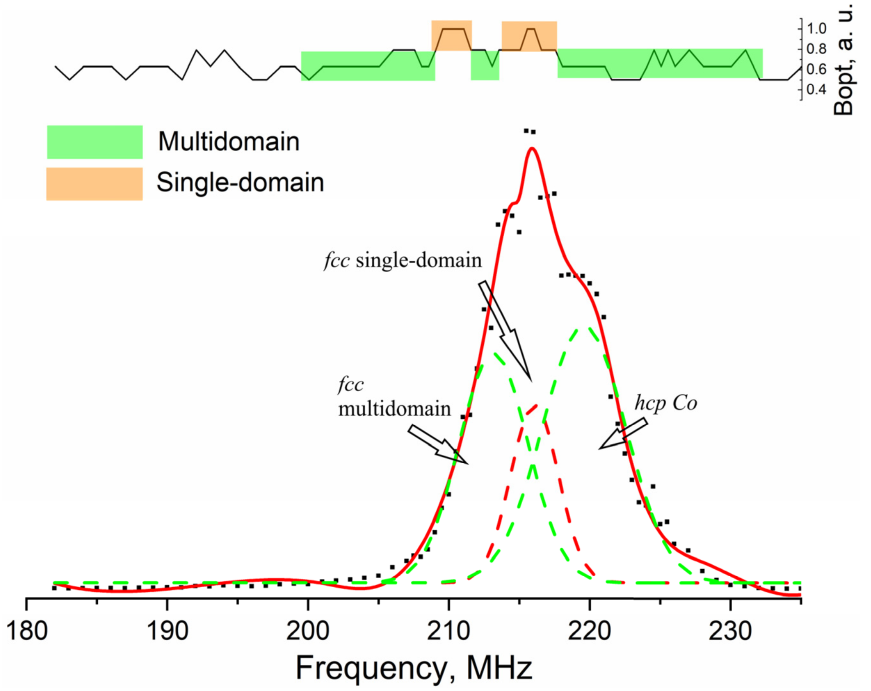

Figure S1. Bottom: decomposition of the ^{59}Co Internal Field NMR spectrum of the hydrogen treated CoZr composite into contributions from different Co structures. Top: optimal excitation magnetic field curve (a. u.). Regions shown in light-green correspond to multidomain particles (low optimal field, high signal enhancement); regions shown in red correspond to single-domain particles (high optimal field, low signal enhancement).
